# ABO blood types associated with the risk of venous thromboembolism in Han Chinese people: A hospital-based study of 200,000 patients

**DOI:** 10.1038/srep42925

**Published:** 2017-03-06

**Authors:** Xuefeng Sun, Jun Feng, Wei Wu, Min Peng, Juhong Shi

**Affiliations:** 1 Department of Respiratory Medicine, Peking Union Medical College Hospital, Beijing, China; 2 Department of Hematology, Peking Union Medical College Hospital, Beijing, China; 3 Department of Clinical Laboratory, Peking Union Medical College Hospital, Beijing, China

## Abstract

ABO blood types are putatively associated with the risk of venous thromboembolism (VTE), but it is not proved in Chinese people. A large population of Han Chinese patients discharged from Peking Union Medical College Hospital between January 2010 and June 2016 were retrospectively analyzed in a case-control study. A total of 1412 VTE patients were identified from 200,660 discharged Han Chinese patients, including 600 patients with deep vein thrombosis (DVT), 441 patients with pulmonary embolism, and 371 patients with both DVT and pulmonary embolism. The prevalence of non-O blood type was weakly but statistically higher in VTE patients compared with 199,248 non-VTE patients, with an odds ratio (OR) of 1.362 (95% confidence interval [CI], 1.205–1.540). Subgroup analysis showed that the OR for non-O blood type was still increased. It was greater in pre-hospital VTE (OR = 1.464) than that in hospital-acquired VTE (OR = 1.224), and greater in unprovoked VTE (OR = 1.859) than that in provoked VTE (OR = 1.227). The OR for non-O blood type decreased with age in subgroup analysis. These results suggest a weak but statistically significant correlation between non-O blood type and risk of VTE in Han Chinese people.

Venous thromboembolism (VTE), which encompasses both deep vein thrombosis (DVT) and pulmonary embolism, is an important cause of morbidity and mortality^1^. Both genetic and environmental factors contribute to the pathogenesis of VTE. Among genetic factors, ABO blood types profoundly influence hemostasis and are putatively associated with VTE^2^,^3^,^4^,^5^. Hospital- and blood donor-based studies have shown that people with non-O blood types have a greater risk of developing VTE than people with type O. This is likely due to the higher blood concentrations of factor VIII and von Willebrand factor in those with non-O blood types^6^,^7^. However, most hospital-based studies have been limited by small sample size, varying quality, and inconsistent outcomes. Hence, a hospital-based study with a large-scale population is imperative to corroborate previous findings.

The genetic profile for thrombosis of Chinese people differs from that of Caucasians. In particular, factor V Leiden and prothrombin G20210A are major risk factors of VTE in Caucasians, but are not detected in Chinese populations^8^. Therefore, it should be investigated whether ABO blood types are associated with the risk of VTE in Chinese people. In this study, we investigated a link between ABO blood types and VTE in a Han Chinese population by referencing a database of more than 200,000 patients who had been discharged from a tertiary hospital in China.

## Results

The data of 200,660 discharged Han patients who had been discharged between January 2010 and June 2016 were collected from the hospital database for this study. ABO blood type was determined from the ABO blood type database for each of these patients. From the Medical Record First Page (MRFP) database, 1443 (0.72%) of these patients had received a diagnosis of VTE. A review of these medical records excluded 31 patients. Therefore, 1412 patients with VTE were included in the case group, consisting of 600 DVT-only patients, 441 pulmonary embolism-only patients, and 371 patients with both DVT and pulmonary embolism. The remaining 199,248 patients were set as the control group. The mean age of all discharged patients was 47.5 years, but patients in the VTE group was much older (mean age, 57.3 years). Men constituted 45.1% of all VTE patients. In regard to precipitating factors, infection, cancer, surgery, arrhythmia, fracture, antiphospholipid syndrome and pregnancy accounted for 41.4%, 32.7%, 22.5%, 6.4%, 4.2%, 3.9% and 0.4% of all VTE patients, respectively.

**Figure 1:**
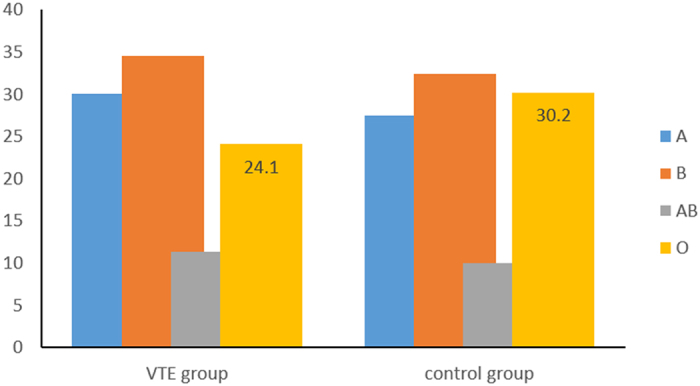
Prevalence of ABO blood types in patients with VTE and in the control group.

Regarding the distribution of blood types, blood type O was the second-most prevalent phenotype (B > O > A > AB; Fig. 1) in non-VTE patients. However, blood type O was the third-most prevalent phenotype in VTE patients (B > A > O > AB).

The prevalence of non-O blood types was statistically higher in VTE patients compared with all the discharged non-VTE patients (Table 1), with an odds ratio (OR) of 1.362 (95% confidence interval [CI], 1.205–1.540). Adjusted OR was unchanged after adjustment for gender and age. Furthermore, in VTE patients when the non-O group was subclassified as blood types A, B, and AB, the ORs between each of these blood types and the O type were comparable to that between the general non-O group and the O group. Pairwise comparisons of the A, B, and AB blood types of the VTE patients and non-VTE patients revealed no statistical differences in ORs.

Subgroup analyses were listed in Table 2. The discharged VTE patients were stratified into DVT and pulmonary embolism subgroups. The prevalence of non-O blood type was statistically higher in both the DVT subgroup (OR = 1.484) and the pulmonary embolism subgroup (OR = 1.288). According to the time when VTE was diagnosed, VTE patients were stratified into patients with pre-hospital VTE and patients with hospital-acquired VTE. The prevalence of non-O blood type was statistically higher in patients with pre-hospital VTE (OR = 1.464) and in patients with hospital-acquired VTE (OR = 1.224). When precipitating factors were considered, VTE patients could be classified into unprovoked VTE and provoked VTE. Both these two subgroups had statistically increased ORs for non-O blood type, but OR was greater in unprovoked VTE patients (OR = 1.859) than that in provoked VTE patients (OR = 1.227). The OR of non-O blood type for VTE was also assessed in discharged male and female patients separately, and the value was unchanged. When VTE patients were stratified based on age, OR for non-O blood type decreased gradually with age, from 1.987 in patients <39 years, to 1.468 in patients 40–59 years, and then 1.157 in patients >60 years. Statistical difference was noted in patients <60 years, but not in patients >60 years. In subgroups with pre-hospital and hospital-acquired VTE cases, and with unprovoked and provoked VTE cases, when the mean ages were compared, a decreased OR was noted in the subgroup with an older mean age.

## Discussion

In this case-control study, we analyzed the distribution of ABO blood types in a large-scale database containing more than 200,000 discharged Han Chinese patients, and found that the prevalence of non-O blood type was statistically higher in patients with VTE compared with non-VTE patients. This suggests that non-O blood types may carry a greater risk of VTE in Han Chinese people. To our best knowledge, this is the largest and probably only study reporting the association between ABO blood types and the risk of VTE in a population with a pure ethnic group.

A multitude of factors, genetic and environmental, contribute to the development of VTE. The ABO blood types have a substantial effect on hemostasis, and previously a number of studies have attempted to prove the association between ABO blood types and risk of developing VTE. Most of the studies were hospital-based or blood donor-based, and had small sample size, varying quality, and inconsistent outcomes. Two meta-analyses performed in 2008 and 2012 both showed increased ORs (1.79 and 2.09, respectively) for non-O blood types in VTE patients compared with non-VTE controls^3^,^9^. Vasan *et al*.^10^ recently revealed an association between ABO blood types and the risk of VTE, based on 1.5 million blood donors. However, blood donors differed from the general population in environmental variables and genetic factors^11^. Therefore, the association between ABO blood types and the risk of VTE still requires verification in other large-scale populations. To our best knowledge, this study is the largest hospital-based study to investigate a correlation between ABO blood types and the risk of VTE, and further provides evidence that non-O blood types increase the risk of VTE compared with blood type O.

When subgroups were analyzed, the most impressive result was that the OR of non-O blood type for VTE decreased with age, not only in discharged patients stratified by age, but also in subgroups with pre-hospital and hospital-acquired VTE cases, and with unprovoked and provoked VTE cases, indicating that age is a confounder in this study. It is already known that ABO blood types are associated with the risk of VTE not only in unprovoked cases^12^, but also in provoked cases, such as cancer^13^,^14^, pregnancy^15^, trauma^16^ or post-surgery^17^,^18^. Meta-analysis showed that the prevalence of non-O blood types is higher in unprovoked VTE cases (OR = 1.88) than in provoked cases (OR = 1.33)^3^. ORs in our present study in regard to precipitating factors were similar to that described in the literature. The finding that ABO blood type was more associated with unprovoked VTE further proves its role as a genetic risk factor. How precipitating factors influence the association between ABO blood types and the risk of VTE are still unknown. Because provoked VTE patients have an older mean age, and age seems to be a confounder in this study, the difference between provoked and unprovoked VTE subgroups may be explained, at least partially, by age. We also found that non-O blood type was associated with VTE more in patients with pre-hospital VTE than that in patients with hospital-acquired VTE. Since hospitalized patients are usually older and have more precipitating factors than pre-hospital cases, this finding seems scientifically understandable.

Individuals with a non-O blood type have higher levels of factor VIII and von Willebrand factor compared with individuals with blood type O^6^,^7^. Elevated factor VIII or von Willebrand factor has been shown to be a moderate risk factor for VTE^19^. Therefore, factor VIII and von Willebrand factor are potentially involved in the correlation between ABO blood types and development of VTE. However, some studies have shown that a non-O blood type carries a risk of VTE, independent of factor VIII^20^,^21^. Other possible mechanisms include the influence of genetic variation in the ABO locus on serum levels of inflammatory markers^22^,^23^,^24^. Hence, the exact mechanisms underlying a higher risk of VTE in non-O blood types are complicated and need further exploration.

In the present study, we noted that the proportion of non-O blood types in the Han Chinese population was about 70%, which is much higher than that of Caucasians (50–60%)^10^,^20^,^25^,^26^. Thus, theoretically a higher proportion of VTE should occur in Chinese people, since non-O blood types are a risk factor. However, on the contrary, incidences of VTE in the Chinese subjects of this study and in the literature were much lower than that reported for Caucasians^27^,^28^,^29^,^30^. Therefore, it can be deduced that some risk factors other than ABO blood types also contribute significantly to the development of VTE in Caucasians. It is already known that the genetic profile for hemostasis and thrombosis in Chinese people is quite different from that of Caucasians^8^, and it is likely that the genetic difference other than ABO blood types between Chinese and Caucasians contributes to the lower incidence of VTE in Chinese. Furthermore, environmental factors such as dietary habit may also contribute to the risk of VTE and cause the different incidence of VTE in different ethnic groups. A similar phenomenon was also observed between Caucasians and African Americans^20^.

In the present investigation, the following measures were taken to enhance the credibility of the results. First, we used the clinical laboratory to obtain ABO blood type data, as the records for blood type were more accurate than those recorded in the MRFP database. Second, in the analysis phase, VTE patients were further analyzed with a multivariable logistic regression model adjusted for gender and age. Besides, five categories of subgroups were analyzed separately. The prevalence of non-O blood type remains unchanged in regression analysis and in subgroup analysis, further confirming the credibility of the results.

It needs attention that although compression venous ultrasonography, computed tomographic pulmonary angiography, and ventilation-perfusion scintigraphy are widely used in the diagnosis of DVT and pulmonary embolism in our hospital, underreporting of VTE patients still may exist in this study. However, such possible underreporting would not be associated with genetic ABO blood types. Thus, VTE patients with non-O and O blood types are equally likely to be underreported. When the actual OR is >1, the OR in the presence of underreporting is less than the actual OR (see Supplementary Fig. S1). In other words, the actual OR should be larger than the OR presented here.

In brief, this study utilized a hospital-based database of more than 200,000 discharged patients to confirm a weak but statistically significant association between ABO blood types and the risk of VTE in a Han Chinese population. The implications of this association and its predictive value in VTE warrant further exploration.

## Methods

### Data sources

Data were retrieved from 2 databases at Peking Union Medical College Hospital, Beijing, China. One is MRFP database, located in the medical records room. For each discharged patient, the MRFP contains the following: name; gender; date of birth; ethnic group; dates and departments of admission and discharge; up to 20 discharge diagnoses based on the International Statistical Classification of Diseases and Related Health Problems Tenth Revision (ICD-10); and surgical procedures. The other database, in the clinical laboratory, contains the ABO blood types of all patients tested for blood type.

### Study design

This was a retrospective case-control study. The main objective was to analyze the association between ABO blood types and the risk of VTE before and during hospitalization. The MRFP database was searched for diagnostic codes indicating DVT or pulmonary embolism in all discharged patients with diagnoses between January 2010 and June 2016. For patients with multiple admissions, only the first was considered. Medical records were then reviewed to verify the diagnosis of VTE. DVT was confirmed by compression venous ultrasonography or computed tomographic venography, and pulmonary embolism was confirmed by computed tomographic pulmonary angiography or ventilation-perfusion scintigraphy. Following details in VTE patients were also retrieved for subgroup analysis: (1) the time when VTE developed; and (2) precipitating factors for VTE, including infection, cancer, surgery, fracture, pregnancy, cardiac arrhythmia, and antiphospholipid syndrome.

Because the distribution of ABO blood type groups differs among ethnicities, in the present study the ethnic group was restricted to Han Chinese, which constitutes 90% of the total population in China. The ABO type of each patient was retrieved from the ABO blood type database. Therefore, the case group consisted of Han Chinese individuals with known blood types, who had received a diagnosis of DVT or pulmonary embolism and who had been discharged from Peking Union Medical College Hospital between January 2010 and June 2016.

The control group consisted of all hospitalized non-VTE Han patients with determined blood types between January 2010 and June 2016; for subjects with multiple admissions, only the first was considered. Age and gender were retrieved for every discharged patient.

The study was approved by the Ethical Committee of Peking Union Medical College Hospital (Reference Number: S-K150). Due to the retrospective nature of the study and no identifying information relating to participants was included, written informed consent was waived. All experiments protocols were conducted according to the Strengthening the Reporting of Observational Studies in Epidemiology guidelines.

### Statistical analysis

Subjects with diagnoses of both DVT and pulmonary embolism contributed to both of the outcome categories. Demographic characteristics were described as mean + standard deviation (SD) for continuous variables and as percentages for categorical variables. Means or prevalence for baseline variables of interest were compared between VTE cases and controls, using independent sample t-tests or chi-squared (χ^2^) tests. ORs with 95% CIs for certain blood types in VTE patients were assessed with logistic regression models. All data were analyzed using the Statistical Package for the Social Sciences software (SPSS, version 19.0; SPSS, Chicago, Illinois, USA). *P*-values < 0.05 were considered statistically significant.

## Additional Information

**How to cite this article**: Sun, X. *et al*. ABO blood types associated with the risk of venous thromboembolism in Han Chinese people: A hospital-based study of 200,000 patients. *Sci. Rep.* **7**, 42925; doi: 10.1038/srep42925 (2017).

**Publisher's note:** Springer Nature remains neutral with regard to jurisdictional claims in published maps and institutional affiliations.

## Supplementary Material

Supplementary Dataset 1

## Figures and Tables

**Table 1:** Demographic characteristics of participants in case and control groups and odds ratios between ABO blood types.

		VTE group	Control group	*P*-value
Subjects, n		1412	199,248	
Male, n (%)		637 (45.1)	77,647 (39.0)	0.000
Age, mean + SD, y		57.3 + 17.3	47.5 + 18.7	0.000
Blood group, n	A	425	54,735	
	B	487	64,490	
	AB	160	19,905	
	O	340	60,118	
Blood group, OR (95% CI)	A - O	1.373 (1.190–1.584)	—	
	B - O	1.335 (1.162–1.534)	—	
	AB - O	1.421 (1.177–1.716)	—	
	Non-O - O	1.362 (1.205–1.540)1.355 (1.199–1.532)*	—	
	A - B	*1.028 (0.902*–*1.172*)	—	
	A - AB	*0.966 (0.805*–*1.159*)	—	
	B - AB	*0.939 (0.785*–*1.124*)	—	

VTE, venous thromboembolism; SD, standard deviation, OR, odds ratio.

^*^OR adjusted for gender and age.

**Table 2:** ORs for Non-O blood type in VTE patients stratified by VTE location, time, precipitating factors, age and gender.

	Subgroups	Age, mean + SD, y	Case group		Control group		OR (95% CI)
			Non-O	O	Non-O	O	
VTE	Total	57.3 + 17.3	1072	340	139,114	60,134	1.362 (1.205–1.540)
	DVT	57.1 + 17.6	752	219	139,114	60,134	1.484 (1.276–1.725)
	PE	57.0 + 17.4	608	204	139,114	60,134	1.288 (1.099–1.510)
	Pre-hospital VTE	56.6 + 17.3	667	197	139,114	60,134	1.464 (1.248–1.716)
	Hospital-acquired VTE	58.7 + 16.4	405	143	139,114	60,134	1.224 (1.011–1.482)
	Unprovoked VTE	53.5 + 18.8	314	73	139,114	60,134	1.859 (1.441–2.399)
	Provoked VTE	58.7 + 16.5	758	267	139,114	60,134	1.227 (1.067–1.411)
	≤39 years	28.6 + 7.1	194	43	47341	20855	1.987 (1.428–2.767)
	40–59 years	50.6 + 5.9	361	105	52455	22391	1.468 (1.180–1.825)
	≥60 years	71.3 + 7.5	517	192	39318	16888	*1.157 (0.979*–*1.366*)
	Male	57.2 + 18.1	483	154	54283	23374	1.351 (1.126–1.620)
	Female	57.4 + 16.6	589	186	84831	36760	1.372 (1.163–1.619)

VTE, venous thromboembolism; DVT, deep vein thrombosis; PE, pulmonary embolism; OR, odds ratio.
