# Supplementary material for: ABO blood types associated with the risk of venous thromboembolism in Han Chinese people: A hospital-based study of 200,000 patients
Source: Sci Rep. 2017 Mar 6;7:42925. doi: 10.1038/srep42925 (PMC5338014; doi:10.1038/srep42925)
Supplement: Supplementary Dataset 1 [file srep42925-s1.doc]

**ABO blood types associated with the risk of venous thromboembolism in Han Chinese people: A hospital-based study of 200,000 patients**

Xuefeng Sun^1+^, Jun Feng^2+^, Wei Wu^3^, Min Peng^1^, and Juhong Shi^1*^

^1^Department of Respiratory Medicine, ^2^Department of Hematology, and ^3^Department of Clinical Laboratory, Peking Union Medical College Hospital, Beijing, China

*[juhong_shi@hotmail.com](mailto:juhong_shi@hotmail.com)


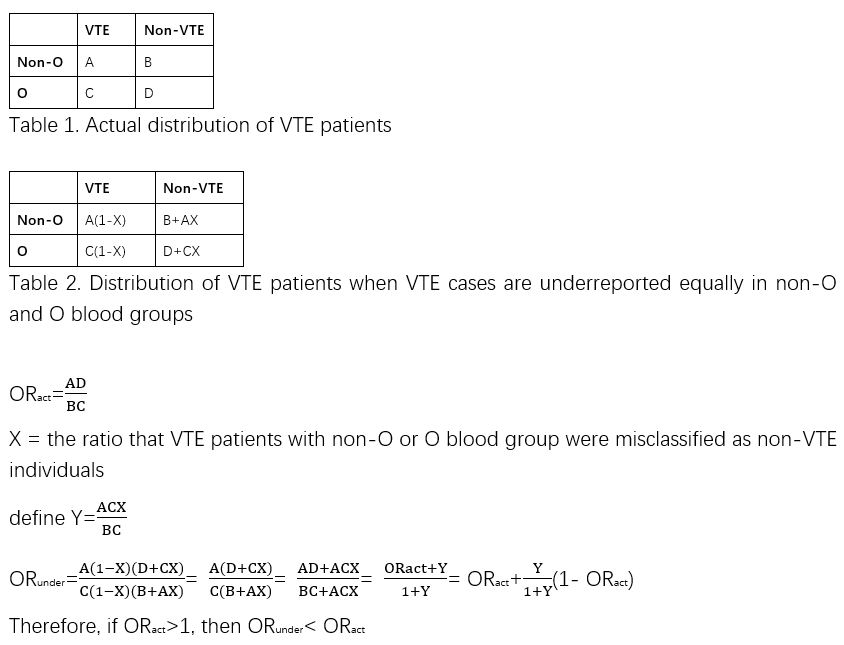


**Figure S1. Detailed calculating course for comparison between actual odds ratio and underreported odds ratio**
